# Functional Role of the C-Terminal Amphipathic Helix 8 of Olfactory Receptors and Other G Protein-Coupled Receptors

**DOI:** 10.3390/ijms17111930

**Published:** 2016-11-18

**Authors:** Takaaki Sato, Takashi Kawasaki, Shouhei Mine, Hiroyoshi Matsumura

**Affiliations:** 1 Biomedical Research Institute, National Institute of Advanced Industrial Science and Technology, 1-8-31 Midorioka, Ikeda, Osaka 563-8577, Japan; takashi-kawasaki@aist.go.jp (T.K.); s-mine@aist.go.jp (S.M.); 2 College of Life Sciences, Ritsumeikan University, Kusatsu, Shiga 525-8577, Japan; h-matsu@fc.ritsumei.ac.jp

**Keywords:** G protein-coupled receptor, olfactory receptor, activation, interaction, Ca^2+^ imaging, response kinetics, homology model

## Abstract

G protein-coupled receptors (GPCRs) transduce various extracellular signals, such as neurotransmitters, hormones, light, and odorous chemicals, into intracellular signals via G protein activation during neurological, cardiovascular, sensory and reproductive signaling. Common and unique features of interactions between GPCRs and specific G proteins are important for structure-based design of drugs in order to treat GPCR-related diseases. Atomic resolution structures of GPCR complexes with G proteins have revealed shared and extensive interactions between the conserved DRY motif and other residues in transmembrane domains 3 (TM3), 5 and 6, and the target G protein C-terminal region. However, the initial interactions formed between GPCRs and their specific G proteins remain unclear. Alanine scanning mutagenesis of the murine olfactory receptor S6 (*m*OR-S6) indicated that the N-terminal acidic residue of helix 8 of *m*OR-S6 is responsible for initial transient and specific interactions with chimeric Gα_15_olf_, resulting in a response that is 2.2-fold more rapid and 1.7-fold more robust than the interaction with Gα_15_. Our mutagenesis analysis indicates that the hydrophobic core buried between helix 8 and TM1–2 of *m*OR-S6 is important for the activation of both Gα_15_olf_ and Gα_15_. This review focuses on the functional role of the C-terminal amphipathic helix 8 based on several recent GPCR studies.

## 1. Introduction

G protein-coupled receptors (GPCRs) form a large protein superfamily comprising nearly 800 members in humans [1]. GPCRs are mainly located in the plasma membrane, where they detect various extracellular physicochemical signals from inside the body or from external environments such as neurotransmitters, hormones, light, and odorous chemicals during neurological, cardiovascular, sensory and reproductive signaling processes via activation of respective target G protein α subunits (Gαs) and their effector proteins for intracellular signals. GPCR signaling systems are involved in many diseases, and some are major therapeutic targets [2]. Due to their abundance and variability, GPCR signaling is highly diverse in terms of ligands, ligand-binding sites, GPCR-specific Gα subunits, and downstream effector proteins. In contrast, the intramolecular interactions underpinning the structural rearrangements of activated GPCRs [3,4] and the essential and extensive interactions between activated GPCR and Gα [5,6] are conserved, at least for class A GPCR signaling. Activation of a specific Gα appears to be mediated by the formation of initial transient and type-specific interactions with activated GPCRs prior to the formation of more stable interactions. This initial transient process can be a potential target for specific GPCR-regulated signaling pathways.

Recently, we found that the second residue of the amphipathic helix 8 in the C-terminal domain of the murine olfactory receptor S6 (*m*OR-S6), a GPCR superfamily member, is responsible for initial transient and specific interactions with chimeric Gα_15_olf_, but not with Gα_15_ [7]. Our mutagenesis analysis also indicates that the hydrophobic core that is buried between the amphipathic helix 8 and transmembrane domains 1–2 (TM1–2) of *m*OR-S6 are important for activation of both Gα_15_olf_ and Gα_15_. In many GPCRs, helix 8 plays several key roles in protein/lipid interaction [8,9], receptor internalization [10], dimerization of receptors [11], and coupling with G proteins [12]. By comparing several GPCRs, this review focuses on the functional roles of the C-terminal amphipathic helix 8 of olfactory receptors (ORs) and other GPCRs.

## 2. A Simple Model of Signal Flow via Interactions in Parallel G Protein-Coupled Receptor (GPCR) Signaling Pathways

GPCR signaling pathways involve the activation of various signaling proteins through key molecular interactions. We considered a simple model of signal flow that proceeds via interactions in parallel GPCR signaling pathways. This is one possible model and does not exclude other mechanisms. In general, the basic principles governing various physicochemical phenomena should be simple and hierarchically assembled, and the molecular mechanisms underpinning the interactions of GPCRs with their ligands or G proteins should be also hierarchically assembled. Intra- and inter-molecular interactions of GPCRs are classified as shared or type-specific across all GPCRs or individual GPCR sub-superfamilies, and interactions are classified as transient or (more) stable. In each step, a specific interaction with a higher binding affinity of a ligand for a receptor is of higher priority. Evidence suggests that initial transient and specific interactions facilitate the shared, extensive (more stable) and partially type-specific interactions [4,5,7], but not vice versa (i.e., shared interactions do not facilitate specific interactions in our model).

GPCRs consist of seven transmembrane-spanning α-helices connected by extracellular loops (EC, including an N-terminus) or intracellular loops (IC, including a C-terminus that contains a short α-helix, helix 8). The concentration of a signaling molecule is highest at its source and gradually increases from nearly zero to a peak value in the vicinity of a GPCR at the membrane of a cell. A given signal molecule is likely to bind to a specific binding site of its target GPCR with a higher affinity than for a nonspecific binding site or to a non-target GPCR. As the concentration of the signaling molecule increases, the agonist-specific interaction results in the target GPCR becoming semi-activated before non-target GPCRs. In order to maintain the activated structural conformation for an adequate time period (being more stable), the initial specific interactions with the agonist facilitate the formation of intramolecular interactions in the activated GPCR, and these are likely to be shared among different GPCR family members. In the next step, the activated GPCR interacts with specific sites or residues of its target G protein, also in an affinity-dependent manner, rather than with non-specific interaction sites of target G proteins or with conserved residues of non-target G proteins. Similarly, in order for semi-activated GPCRs to form extensive interactions with their target G proteins for an adequate period of time to allow the exchange of guanosine 5′-diphosphate (GDP) for guanosine triphosphate (GTP), a second set of specific and transient interactions presumably facilitate the formation of the GPCR–G protein complex in a fully activated form, and these more stable intermolecular interactions are likely to be shared, at least in part, between different GPCRs. In parallel GPCR signaling pathways, specific activation sequences enable target GPCRs to first detect signaling molecules and activate target G proteins, and also to initiate activation or inactivation of specific effector proteins to bring about the desired function. This avoids functional disorders resulting from stimulation or inactivation of non-target effector proteins. This functional model allows us to predict intra- and inter-molecular interactions, as follows:

(1) An agonist molecule binds to a specific binding site of a target GPCR (first semi-activation).
(2) Binding of a specific agonist induces the structural rearrangement of GPCR transmembrane domains, leading to conformational changes and transition to an active state (activation by an agonist).
(3) The activated GPCR initially and transiently interacts with a target heterotrimeric G protein [7] comprised of α-, β- and γ-subunits (second semi-activation).
(4) In the initial and transient interaction between the GPCR and a semi-activated target G protein, displacement of helix-α5 of the Gα subunit towards TM3 of the GPCR facilitates the formation of a more stable, ternary activated GPCR–heterotrimeric G protein complex that is mediated by shared and/or partially specific molecular interactions [5] (full activation).
(5) In the stable, ternary activated GPCR–heterotrimeric G protein complex, the Gα subunit releases GDP from the binding pocket.
(6) A GTP then binds to the nucleotide-free Gα subunit, followed by dissociation of Gα and βγ subunits from the GPCR [5].
(7) The Gα and βγ subunits interact with their respective effector proteins, thereby regulating their activities in the process.

As described above, steps (1) and (3) are likely to be specific to a target GPCR or G protein, whereas steps (2) and (4) are likely to be shared between different GPCRs and G proteins.

## 3. Structural Features of Helix 8

**Figure 1:**
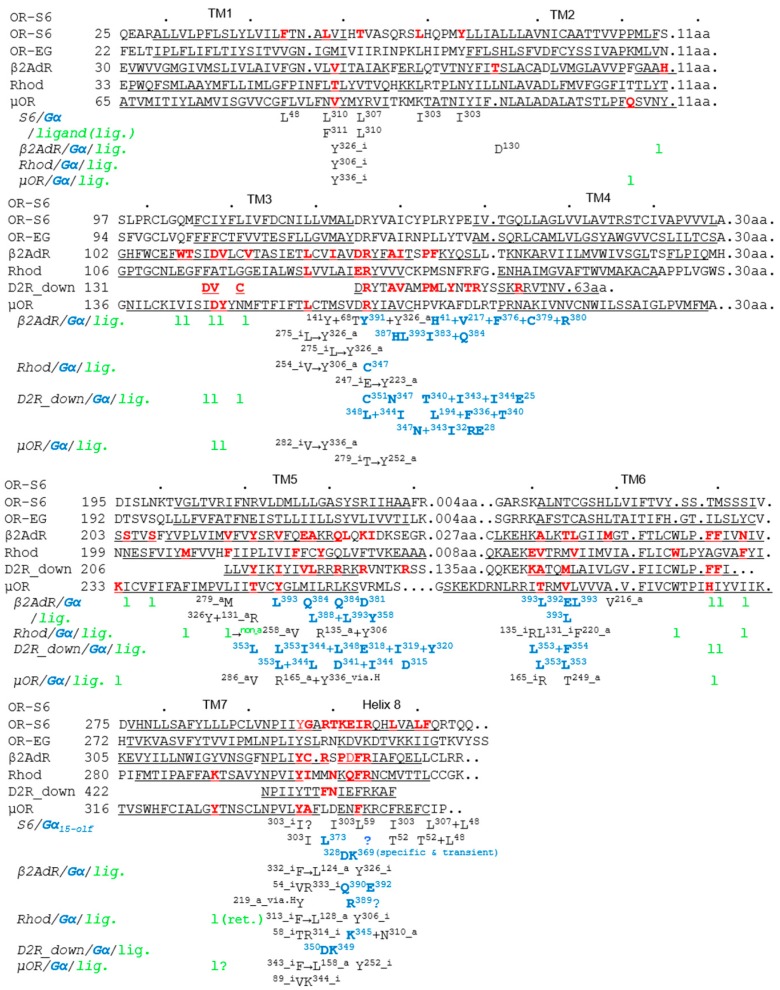
Multiple alignment and interacting residues of the transmembrane domains (TMs) and helix 8 of mammalian class A G protein-coupled receptors (GPCRs). The predicted secondary structure of the murine olfactory receptor S6 (OR-S6, top, *Mus musculus*, UniProt accession number: Q9WU88, Reference [7] with permission for authors), eugenol olfactory receptor (OR-EG, *Mus musculus*, Q920P2, Reference [7]) secondary structures from β_2_ adrenergic receptor (β_2_AdR, *Homo sapiens*, P07550, Reference [21]), rhodopsin (Rhod, *Bos taurus*, P02699, Reference [22]), dopaminergic D2 receptor with the side chain of His^393^ pointing toward the intracellular part of the receptor (D2R_down, Reference [6]) and µ-opioid receptor (µOR, bottom, *Mus musculus*, PDB id: 4DKL, Reference [3]) are shown. Underlined sequences indicate α-helices. In the lower rows, intra- and inter-molecular interaction residues (superscripts indicate the position number of the amino acid; _i, inactive state; _a, active state; non, no interactive ligand or residue; via.H, through a water-mediated polar network, References [3,4,5]) of GPCRs (black), G protein α-subunits (blue bold) and ligands (green l) are shown below the interacting residues (red bold) of each GPCR. In the case of OR-S6, residues were predicted by our homology model or from the results of other GPCRs as described in the main text. The amino acid sequence number (top, every 10th residue marked with dots) and the total number of fragments (right) of murine OR-S6 are shown.

The most important structural feature of helix 8 is an amphipathic short α-helix. In rhodopsin (Rhod), helix 8 acts as a membrane surface recognition domain, and adopts a helical structure only in the presence of membranes or membrane mimetics [9]. Helix 8 begins after a short linker following TM7, at the end of which the conserved NPxxY motif is located, as shown in a selection of class A GPCR sequences (Figure 1) [3,4,5,6,7]. The short linker between TM7 and helix 8 is also important, as described in Section 5 below. Crystal structures revealed that helix 8 lies parallel to the membrane in both inactive and active states in the β_2_ adrenergic receptor (β_2_AdR) and in Rhod [5,13,14,15]. Moreover, in the inactive state of these GPCRs, the third residue (Phe) of helix 8 interacts with the Tyr residue of the NPxxY motif in TM7 [5,15], and mutation within this motif causes a significant reduction in signaling activity [15,16]. The third residue of helix 8 is commonly hydrophobic (Phe, Ile, Val, etc.) in mammalian class A GPCRs, including ORs that comprise the largest superfamily [17]. In the inactive state of GPCR, the Tyr-Phe/Ile/Val intramolecular interaction forms part of the hydrophobic core between helix 8 and TM1–2 [5,15], which stabilizes the orientation and position of the N-terminal region of helix 8 [7].

**Figure 2:**
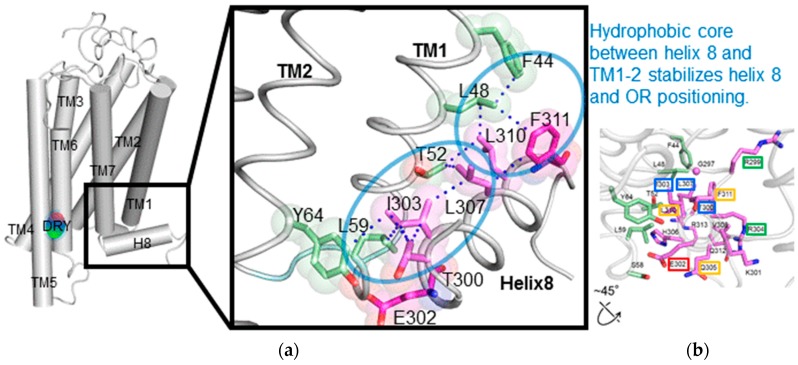
Model of *m*OR-S6 generated by homology modeling (modified from Reference [7] with permission for authors). (**a**) The overall model of *m*OR-S6 (left). The right figure represents an enlarged view of the region surrounding helix 8 that includes functionally important residues that have been experimentally investigated. Residues involved in hydrophobic interactions surrounding helix 8 are shown as transparent CPK spheres and labeled accordingly. Residues of helix 8 are colored magenta, while TM1 and TM2 residues are green; (**b**) Detailed interfaces of helix 8 and TM1–2 rotated 45° from the view shown in the top panels. Residues involved in hydrophobic interactions surrounding helix 8 and Glu^302^ are shown in stick representation.

**Figure 3:**
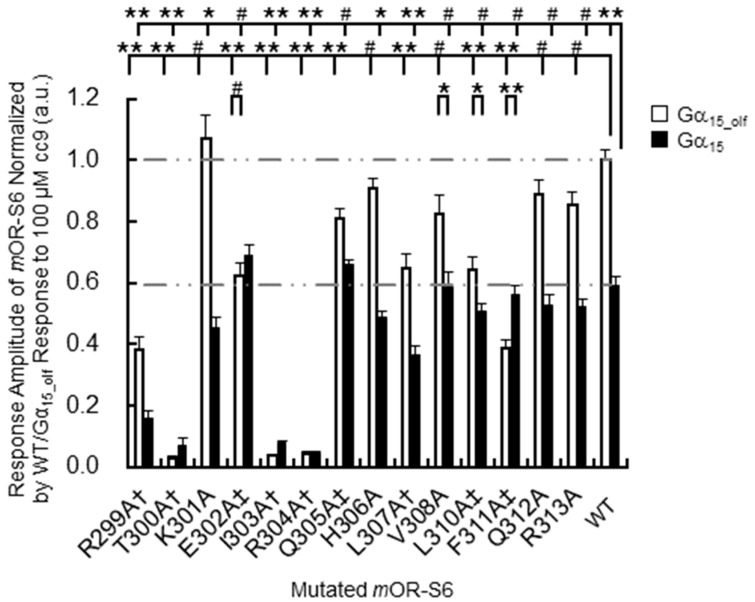
Scanning alanine mutations of *m*OR-S6 helix 8 and their associated calcium responses in a heterologous HEK293 system based in the average Ca^2+^ responses to cc9 (from Reference [7] with permission for authors). Error bars indicate standard errors of the mean. Indiscriminate inactivation via Gα_15_olf_ or Gα_15_ is indicated by † and selective inactivation via Gα_15_olf_ or Gα_15_, is indicated by ‡. Statistical significance was determined by the *t*-test and is labeled at the top of each bar (# *p* ≥ 0.01; 0.001 ≤ * *p* < 0.01; ** *p* < 0.001).

To investigate the structural details of *m*OR-S6 using alanine scanning mutagenesis, a model for the 3D structure was constructed by homology modeling (Figure 2) [7] with crystal structures of active Rhod (the Protein Data Bank (PDB) id 3PQR) and β_2_AdR (PDB id 3SN6) as templates. The best model was chosen based on the discrete optimized protein energy (DOPE) score (statistical score derived from atom pairing frequencies in the PDB) using MODELLER 9.1 [18], and the model was further validated by PROCHECK (ver. 3.5) [19]. In both Rhod and β_2_AdR structures, the C-terminal amphipathic helix 8 is stabilized by a hydrophobic core on the intracellular side of the membrane [12,13]. Similarly, in our homology model, the hydrophobic core of both the N-terminal linker (Thr^300^) and helix 8 (Ile^303^, Leu^307^, Val^308^, Leu^310^ and Phe^311^) of *m*OR-S6 are surrounded by TM1 (Phe^44^, Leu^48^, and Thr^52^), IL1 (Leu^59^) and TM2 (Tyr^64^). The hydrophobic residues of helix 8 can be categorized into two groups: The first group contains Thr^300^, Ile^303^, and Leu^307^, which are located at the N-terminal side and the middle region of helix 8. These residues play a crucial role in appropriately positioning helix 8, and mutation of these residues likely disrupt the hydrophobic core and prevent activation of Gα. Indeed, in our alanine-scanning mutagenesis analysis of helix 8, mutation of I303A in *m*OR-S6, equivalent to Phe^332^ in β_2_AdR, caused a drastic decrease in agonist-induced Ca^2+^ responses in HEK293 cells (Figure 3) [7]. This result indicates that disruption of the hydrophobic interactions between Ile^303^ and Thr^300^ (N-terminal linker), Leu^307^ (helix 8) and Leu^59^ (IL1) lead to impaired activation of Gα by *m*OR-S6. The third residue of helix 8, Ile^303^ in *m*OR-S6, appears to be essential for stabilizing the structure of helix 8, and it is also essential for Gα activation along with Thr^300^, the last residue of the N-terminal linker region. Alanine mutations T300A and L307A led to drastic and moderate decreases in Ca^2+^ responses, respectively, since the effect of mutating the N-terminus was greater than that of the middle region (Figure 2 and Figure 3) [7]. Scanning mutagenesis of the M1 muscarinic acetylcholine receptor (M1R, specific to G_q_, a member of the G_q/11_) similarly indicated that hydrophobic core residues are functionally important [20].

In order to identify GPCR residues responsible for specific interaction with Gα, we investigated the kinetics of agonist-induced cellular Ca^2+^ responses of *m*OR-S6 by comparing chimeric Gα_15_olf_ with Gα_15_. The chimeric Gα_15_olf_ has the Gα_olf_ (a member of the G_s_ class) C-terminal ^376^KQYE motif instead of the corresponding ^369^DEIN sequence present in Gα_15_ (a member of the G_q/11_ class), and this change improves the rapidity of the response (2.2-fold shorter Ca^2+^ response onset latency) as well as the response amplitude (1.7-fold), compared with Gα_15_ [7,23]. As expected, EC_50_ values for the most potent agonist of *m*OR-S6 showed no significant difference between Gα_15_olf_ and Gα_15_ [23]. These results indicate that the observed improvements in kinetics are likely attributable to specific interactions at the C-terminal region of Gα_15_olf_ with ORs. In β_2_AdR, Arg^131^ of the DRY motif is packed against the fourth residue (Tyr^391^) of the C-terminal region of Gα_s_ [5]. This intimate interaction between *m*OR-S6 DRY-motif Arg^126^ and Gα_15_olf_ C-terminal fourth Tyr^371^ is believed to be responsible for the rapid and robust responses of ORs with Gα_15_olf_. During the initial interaction step, conformational heterogeneity of TM7 in agonist-bound β_2_AdR [24] may facilitate interactions between the Gα C-terminal domain and the GPCR TM7 helix 8. Further kinetic analysis to unequivocally define the residues responsible for the specific interactions between GPCRs and G proteins are discussed in Section 5 below.

The second group of *m*OR-S6 helix 8 hydrophobic residues includes Leu^310^ and Phe^311^, located at the C-terminal interface between helix 8 and TM1. Alanine mutants L310A and F311A of *m*OR-S6 caused moderate and dramatic decreases in Ca^2+^ responses with Gα_15_olf_, respectively, compared with *m*OR-S6 (Figure 3). Weakening of the hydrophobic core in the vicinity of the helix 8 C-terminal region likely increases helix 8 flexibility and destabilizes its structure. This suggests that activation of Gα_15_olf_ requires a solid and stable helix 8, whereas activation of Gα_15_ does not have this requirement. In total, seven and five of helix-8 alanine mutants reduced the signaling of *m*OR-S6 via Gα_15_olf_ and Gα_15_, respectively (Figure 3). Immunostaining of N-terminal rhodopsin-tagged *m*OR-S6 with anti-rhodopsin antibody confirmed that all *m*OR-S6 mutants were efficiently expressed and membrane-localized [7].

## 4. Shared Features of Different GPCR–G Protein Interactions in Inactive and Active States

In the intra- and inter-molecular interactions of GPCRs that occur during activation and inactivation, the relative position of conserved motifs and residues is clearly important. Key residues controlling GPCR–G protein coupling are believed to be located at the intracellular end of TM5, the N-terminal region of intracellular loop 3 (IL3), the junction of TM3 (including the DRY motif) and IL2, the C-terminal TM6, and the junction of TM7 and helix 8 [4,5,6,21,22]. Comparison of structural differences between inactive and active states of class A GPCRs indicates shared features in the structural rearrangement of activated GPCRs (Figure 1) [3,4]. In β_2_AdR (specific to G_s_, a member of the G_s_ class), Rhod (specific to G_t_, a member of the G_i/o_ class), μ-opioid receptor (µOR, specific to G_i_, a member of the G_i/o_ class) and M2 muscarinic receptor (M2R, specific to G_i_), Arg3.50 of the DRY motif interacts with either Glu6.30 or Thr6.34 in the inactive states (except for M2R and β_2_AdR), but similarly with Tyr5.58 in all active states (*x.yz* numbers follow the Ballesteros-Weinstein numbering method for GPCRs [25]) (Figure 1, Arg^131^–none → Tyr^219^ in β_2_AdR; Arg^135^–Glu^247^ → Tyr^223^ in Rhod; Arg^165^–Thr^279^ → Tyr^252^ in µOR) [3]. Tyr7.53 of the NPxxY motif and the adjacent Cys/Ile/Ala7.54 also interact with the third (Phe8.50) and fourth (Arg/Lys8.51) residues of helix 8 respectively, in the inactive states [4,14,15], whereas Tyr7.53 interacts with Tyr5.58 and Leu3.43 through a water-mediated polar network in active state structures (Figure 1, Tyr^326^–Phe^332^ → Tyr^219^ + Leu^124^ and Cys^327^–Arg^333^ → none in β_2_AdR; Tyr^306^–Phe^313^ → Tyr^223^ + Leu^128^ and Ile^307^–Arg^314^ → none in Rhod; Tyr^336^–Phe^343^ → Tyr^252^ + Leu^158^ and Ala^337^–Lys^344^ → none in µOR) [3]. In our scanning mutagenesis analysis, mutation of the *m*OR-S6 helix-8 fourth residue (R8.51A) resulted in a drastic decrease in Ca^2+^ responses for both Gα_15_olf_ and Gα_15_ (Figure 3) [7]. This is consistent with previous reports that β_2_AdR Arg8.51^333^ [6] and β_1_ adrenergic receptor Arg8.51^384^ [12] are essential for coupling with G proteins. Notably, recent analysis has indicated that Ile/Leu/Met3.46 interacts with Leu/Val/Ile6.37 in inactive states, but with Tyr7.53 in active states (Figure 1, Ile^127^–Leu^275^ → Tyr^326^ in β_2_AdR; Leu^131^–Val^254^ → Tyr^306^ in Rhod; Met^161^–Val^282^ → Tyr^336^ in µOR) [4].

A key feature of β_2_AdR activation is the ~14 Å outward movement of the intracellular portion of TM6, creating a cavity large enough to accommodate the C-terminus of Gα [5,26]. The active state of β_2_AdR is stabilized by extensive interactions with Gα [5]. In an atomic resolution structure of the β_2_AdR-G_s_ complex, the essential and stable interface buried between activated β_2_AdR and Gα_s_ is formed by IL2, TM5 and TM6 of β_2_AdR and by helix-α5, the αN-β1 junction, the top of strand β3 strand, and helix-α4 of Gα_s_ [5]. Among the most conserved amino acids, β_2_AdR Arg^131^ (TM3 DRY motif) is packed against both Gα_s_ Tyr^391^ (helix-α5, fourth residue from the C-terminus of Gα_s_) and β_2_AdR Tyr^326^ (TM7 NPxxY motif) [5]. β_2_AdR Leu^275^ (TM6) also interacts with Gα_s_ Leu^393^ (the penultimate residue for the C-terminus) [5,26]. In addition, β_2_AdR Phe^139^ (IL2) docks into a hydrophobic pocket formed by Gα_s_ His^41^ (β1-strand), Val^217^ (β3-strand), Phe^376^ (helix-α5), Cys^379^ (helix-α5), Arg^380^ (helix-α5) and Ile^383^ (helix-α5) [5]. The position of Phe^139^ (IL2) is stabilized by interactions between Asp^130^ (DRY motif) and Tyr^141^ (IL2) [5]. Notably, the residue corresponding to Phe^139^ is a Phe or Leu in almost all G_s_-coupled GPCRs [5]. In the crystal structure of Rhod in complex with the Gα_t_ C-terminal peptide (GαCT2), the Rhod D(E)RY motif Arg^135^ forms a hydrogen bond to the backbone carbonyl oxygen at the fourth residue from the C-terminus of GαCT2 in the C347V mutant [22], which is similar to the packing of the β_2_AdR DRY motif Arg^131^ against Gα_s_ Tyr^391^. However, rather than Arg^135^, Rhod D(E)RY motif Glu^134^ binds to NPxxY motif Asn^302^ via a water-mediated polar network [22].

The results of a solution-state nuclear magnetic resonance (NMR) study raised the possibility that the propagation of conformational changes in GPCRs occurs via initial interactions between GPCR helix 8 and the associated G protein [27]. Using C^13^-dimethylated µOR, NMR spectroscopy revealed that the agonist-induced spectral changes in helix 8 (Lys8.51^344^) and IC1 (Lys^98^, Lys^100^) were larger than those of TM6 (Lys6.24^269^/Lys6.26^271^) and TM5 (Lys5.66^260^). Interestingly, the presence of both an agonist and a Gα_i_-mimetic nanobody resulted in a complete loss in the intensity of peaks corresponding to helix 8 and the IC1 Lys residue, and a drastic reduction in the intensity of the TM6 Lys peak, with a concomitant appearance of new intense peak. The spectral shift in the TM6 Lys peak presumably reflects the >10 Å outward movement of TM6 in the active state. Sharp, narrow intense peaks for TM6 and TM5 Lys residues indicate a relatively stable conformation for these features, while broad and irregular peaks for helix 8 and IC1 suggest that two or more conformations of helix 8 and IC1 exchange on a low ms time scale in µOR in the presence of agonist alone [27]. In contrast to the relatively stable positions of TM6 and TM5, helix 8 is likely to be more flexible and thus able to adopt the required conformations for forming specific interactions with Gα C-terminal residues, as will be described in the next section.

## 5. Helix 8 N-Terminal Residues of GPCRs Are Responsible for Rapid Kinetics Associated with Specific G Protein Activation

**Figure 4:**
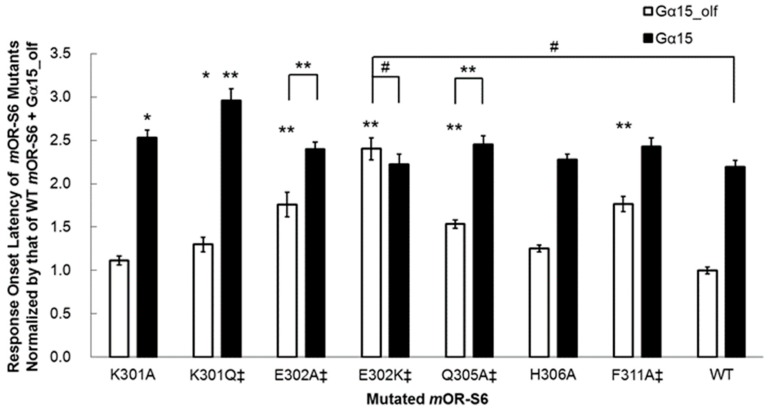
Response onset latency is markedly increased for Gα_15_olf_ but not for Gα_15_ in in *m*OR-S6 E302K (from Reference [7] with permission for authors). The average Ca^2+^ responses to cc9 are shown for *m*OR-S6 helix 8 mutants. Selective inactivation via Gα_15_olf_ or Gα_15_ is indicated by ‡. Statistical significance was determined by the *t*-test and is labeled at the top of each bar (# *p* ≥ 0.01; 0.001 ≤ * *p* < 0.01; ** *p* < 0.001).

Establishing which residues of GPCRs are responsible for the specific interactions with G proteins has received considerable attention. Chimeric mutants of Rhod in which ^300^NKQ is replaced with the ^330^SPD sequence of β_2_AdR (the middle of which is the first amino acid of helix 8) displayed a dramatic decrease in the ability to activate the target G_t_ [28,29]. Furthermore, we examined the contribution made by each residue of helix 8 of *m*OR-S6 to the response kinetics using alanine-scanning mutagenesis. Four mutations (E302A, Q305A, L310A, and F311A) caused a decrease in agonist-induced Ca^2+^ responses in HEK293 cells via Gα_15_olf_, but not via Gα_15_ (Figure 3) [7]. Of these four residues, only mutation of Glu^302^ to alanine resulted in no significant difference in the amplitude of the Ca^2+^ response between Gα_15_olf_ and Gα_15_, but a significant difference in response onset latency was still apparent (Figure 4) [7]. Interestingly, this second residue of helix 8 is negatively charged (Glu or Asp) or uncharged, but is polar (Gln) in the OR family (Table 1).

We examined the effect of introducing a positively charged residue in the E302K mutant, and the improved kinetics of the onset latency and amplitude with Gα_15_olf_ were completely abolished in this variant, which showed no significant differences from wild-type *m*OR-S6 with Gα_15_ (Figure 4) [7]. These results suggest that the N-terminal acidic residue of helix 8 of an OR is responsible for rapid activation of Gα_15_olf_. In the crystal structure of the opsin–Gα_t_ C-terminal peptide (GαCT) complex, the second residue of helix 8 (Gln^312^) interacts with the sixth residue from the C-terminus of Gα_t_ (Lys^345^) and the opsin helix-8 N-terminal linker residue Asn^310^, in addition to the interaction between the opsin D(E)RY motif Arg^135^ and the fourth residue from the C-terminus of Gα_t_ (Cys^347^) (Figure 1) [30]. Molecular modeling revealed differences between intermediary (R*–G_t_(GDP) complex) and stable (R*–G_t_(empty) complex) interactions [31]. Specifically, the second residue of helix 8 of Rhod (Gln^312^) interacts in an intermediate manner with the fourth residue from the C-terminus of Gα_t_ (Cys^347^), but then stably interacts with the sixth residue from the C-terminus (Lys^345^). However, in the crystal structures of the Rhod–GαCT2 complex and the stable β_2_AdR–G_s_ complex, no such interaction between the second residue of helix 8 and Gα was observed. This difference is likely attributable to the C347V mutation of GαCT2 and the stable (i.e., not intermediate) active state of β_2_AdR, respectively. Taken together, these observations indicate that the initial transient and specific interaction between the second residue of *m*OR-S6 helix 8 (Glu^302^; Gln^312^ in opsin) and the sixth residue from the C-terminus of Gα_15_olf_ (Lys^369^; Lys^345^ in Gα_t_) likely facilitates the rapid formation of the active state in the OR–Gα_15_olf_ complex, but not in the OR–Gα_15_ complex. If this is the case, the question arises as to which residues of *m*OR-S6 initially interact with Gα_15_.

Notably, the KE301-302EK double mutant of *m*OR-S6 exhibits an impaired Ca^2+^ response via both Gα_15_olf_ and Gα_15_ (our unpublished data). Moreover, mutation of the first residue of helix 8 of *m*OR-S6 (Lys^301^), which is conserved in the OR family, resulted in mutants that displayed a complicated behavior [7]. The K301A mutation resulted in a significant, but not drastic, decrease in Ca^2+^ responses via Gα_15_ but no change in the responses via Gα_15_olf_ (Figure 3). Meanwhile, the K301A mutation delayed the onset latency, consistent with the decreased response via Gα_15_ but no change via Gα_15_olf_ (Figure 4). Mutation to an uncharged polar residue (K301Q) resulted in similar changes to K301A in terms of response kinetics. However, in contrast to the KE301-302EK double mutant and the K301A/Q mutants, the K301E single mutant with a negatively charged residue, displayed a drastic and selective decrease in response to Gα_15_, but not for Gα_15_olf_. These results raised the possibility that Lys^301^ may attract a negatively charged region of Gα_15_, but not necessarily for Gα_15_olf_. Based on sequence differences between Gα_15_olf_^369^KQYE and Gα_15_^369^DEIN, Gα_15_ Asp^369^ and/or Glu^370^ might be involved in such an initial attraction. In the M3 muscarinic acetylcholine receptor (M3R, specific to G_q/11_), an agonist-induced increase in disulfide cross-linking of the first residue of helix 8 (via the K548C mutant) and the α4/β6 loop of G_q_ (via the D321C mutant) was observed, and was greatly reduced by the pretreatment of membranes with the inverse agonist, atropine [32]. This indicates an interaction between M3R helix 8 Lys^548^ and G_q_ α4/β6 loop Asp^321^. Similarly, *m*OR-S6 helix 8 Lys^301^ may interact with Gα_15_ α4/β6 loop Asp^328^ with slower response kinetics than the inter-helix interaction between *m*OR-S6 and Gα_15_olf_, while the K301E mutation may impair the interaction with Gα_15_ and hence decrease its activation. Thus, kinetic analysis is very useful for evaluating specific interactions between GPCRs and G proteins.

As described above, transient interactions between the second residue of *m*OR-S6 helix 8 (Glu^302^) and the sixth residue from the C-terminus of Gα_15_olf_ (Lys^369^) likely facilitate the rapid formation of a more stable and active OR–Gα_15_olf_ complex, resulting in a rapid and robust Ca^2+^ response. If this is the case, the question arises as to exactly how *m*OR-S6 helix 8 accommodates the Gα α5 C-terminal region between TM3 and TM5 in the stable and active state. Considering the simplest case of β_2_AdR (*m*OR-S6) and its relative movement toward Gα, the C-terminus of Gα α5 may forward towards the N-terminal region of β_2_AdR (*m*OR-S6) helix 8 under TM domain assembly from the intracellular spacing between TM3 and TM5. This relative movement is likely the trigger for an outward movement of the intracellular portion of TM6 that resides on the front side of the N-terminus of helix 8 and may be ready to move following rearrangement of the TM domains upon agonist binding to β_2_AdR. A forward movement of the C-terminal region of Gα α5 would then promote its docking onto the N-terminus of β_2_AdR (*m*OR-S6) helix 8, resulting in the formation of a specific interaction between the sixth residue from the C-terminus of Gα_s_ (Arg^389^ in helix-α5; Lys^369^ of Gα_15_olf_) and the second residue of β_2_AdR helix 8 (Asp^331^; Glu^302^ in *m*OR-S6) at the corner of helix 8 and the membrane surface, rather than at the open surface of helix 8 (the first residue of this region, Figure 2). This step also facilitates the breakage of the interaction between the NPxxY motif Tyr7.58^326^ (Tyr7.58^296^ of *m*OR-S6) and the third residue of helix 8 (Phe8.50^332^; Ile8.50^304^? of *m*OR-S6), which is caused by the outward movement of the adjacent Asp^331^ due to the forward momentum of the transiently interacting C-terminal region of Gα. This presumably results in the movement of helix 8 and Gα C-terminus being pushed back towards TM3 through intra-TM interactions that underpin the elastic properties. This likely results in intimate interactions between β_2_AdR TM3 DRY-motif Arg^131^ and both the fourth residue from the C-terminus of Gα_s_ (Tyr^391^) and β_2_AdR NPxxY motif Tyr^326^, which stabilizes the active state of the ternary complex [5]. These proposed transient perturbations of helix 8 are consistent with the moderately dynamic conformational changes observed for C^13^-dimethylated µOR [27].

This model also explains the greater selective decrease in the Ca^2+^ response for Gα_15_olf_ observed in *m*OR-S6 F311A compared with the L310A mutant, since weakening of the hydrophobic core at the C-terminus of helix 8 likely increases its flexibility and destabilizes the position of Glu^302^ between the membrane and the open surface to a greater extent than disruption of the hydrophobic core within the middle of helix 8. This model, therefore, offers a possible explanation for the rapid formation of a more stable ternary GPCR–G protein complex. Truncated mutants provide further support that helix 8 is essential in GPCR signaling [7,33].

Notably, molecular dynamics simulation and mutagenesis studies of the cannabinoid 1 (CB1, specific to G_i_) receptor suggested that Arg^400^ (the penultimate amino acid of the N-terminal linker) interacts with the penultimate residue of Gα_i_ (Leu^353^) [34]. The penultimate Leu^393^ point mutation to Ala in Gα_s_ also reduced the activity of both β_2_AdR and luteinizing hormone receptor (LHR) [35]. Furthermore, in our scanning mutagenesis analysis, two mutations of this CB1 Arg^400^ equivalent (R299A and R299E), respectively, markedly reduced and completely ablated the Ca^2+^ response [7]. These results suggest that the penultimate residue of the N-terminal linker between helix 8 and TM7 might be responsible for the recruitment of the G protein C-terminus.

## 6. The Second Residue of Helix 8 Partially Governs the Hierarchy of GPCR-Associated Information in Parallel GPCR Signaling Pathways

The replacement of Gα_15_^369^DEIN with Gα_olf_^376^KQYE improved the response kinetics of *m*OR-S6 via the chimeric Gα_15_olf_ by shortening the onset latency 2.2-fold [7,23], but replacement of *m*OR-S6 Glu^302^ with Arg^302^ completely eliminated the effect of this mutation on *m*OR-S6-mediated Ca^2+^ responses [7]. These findings clearly indicate that the second residue of helix 8 is a major determinant of the initial specific interaction with the target G protein that are essential for a rapid and robust response, rather than with Arg or Ala at the second position of helix 8 in ORs or non-target G proteins. In the olfactory system, the second residue of helix 8 appears to govern the sensory processing hierarchy of elemental odors that are represented in the third-order neurons of olfactory pathways [36,37,38,39].

**Figure 5:**
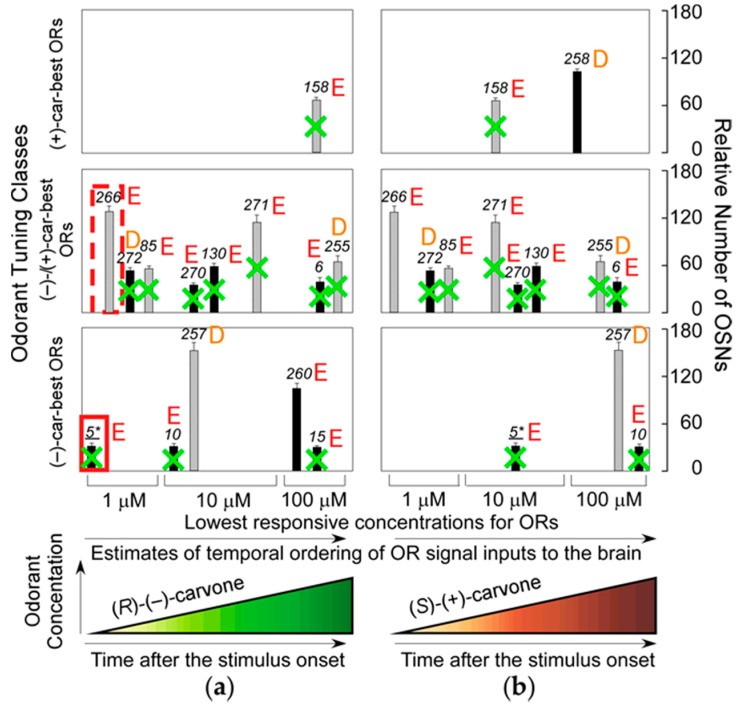
Estimated temporal ordering of input signals from carvone ORs to the brain in ΔD mice (modified from Reference [36] with permission for authors). (**a**) Temporally ordered signal inputs to the brain and the relative number of olfactory sensory neurons (OSNs) expressing the (*R*)-(−)-carvone-activated OR are shown. In wild-type mice (including dorsal ORs marked by green bold crosses), the most sensitive dorsal ORs enhance (*R*)-(−)-carvone-unique elemental odors in the brain by selective summation of cognate OR signals via synchronized inputs to the third-order neurons through feedforward inhibition driven by signals from the most sensitive helix-8-second-Glu dorsal ORs, one of which is enclosed by the red rectangle; (**b**) Temporally ordered signal inputs to the brain and the relative number of OSNs expressing the (*S*)-(+)-carvone-activated OR. In contrast, in ΔD mice lacking dorsal ORs, indicated by green bold crosses, these are the most sensitive common ORs, one of which is enclosed by the red broken-lined rectangle. These govern the prominent elemental odors in the brain. Numbers represent the identities of OR. Among the 15 identified carvone-ORs, 11 are helix-8-second-Glu ORs (marked by E) and four are helix-8-second-Asp ORs (marked by D). In each tuning class ((−)-car-best, (*R*)-(−)-carvone-best; (−)-/(+)-car-best, (*R*)-(−)-/(*S*)-(+)-carvone-best; (+)-car-best, (*S*)-(+)-carvone-best), the most sensitive ORs are all of the helix-8-second-Glu type. Input orders are based on the OR sensitivity and relative response amplitude. The number of in situ hybridized olfactory sensory neurons (indicated by the hatched bars) may be overestimated by potential cross-reactions with other ORs sharing >85% sequence homology.

We proposed a mechanism for supersensitive odor discrimination wherein signals from the helix 8 second residue Glu of dorsal ORs determines the most prominent elemental odor of a given odorant [36]. In odor detection/discrimination behavioral assays, wild-type mice can discriminate similar odors of enantiomeric pairs at sub-ppq (<10^−15^) level, which equates to supersensitivity for enantiomer detection, whereas transgenic mice in which all dorsal ORs are ablated display a >10^10^-fold reduction in enantiomer discrimination sensitivity, although the supersensitive detection capability for (−)-enantiomers is retained [37]. This result indicates that the most sensitive ORs that enable the transgenic mice to detect (−)-enantiomers but not (+)-enantiomers at sub-ppq level do not allow the mice to discriminate (−) from (+)-enantiomers with supersensitivity (odor discrimination paradox), and suggests that some of the most sensitive ORs ablated may enhance characteristic elemental odors in wild-type mice. Among the ablated dorsal ORs with a Glu in the second position of helix 8, *m*OR-*car-c5* is one of the most sensitive and specific for (*R*)-(−)-carvone (Figure 5). These results indicate that the highly sensitive helix-8-second-Glu dorsal ORs play a critical role in hierarchical elemental odor coding by summating synchronized inputs from cognate ORs to third-order neurons for elemental odors through feedforward inhibition [37].

The hierarchical odor-coding hypothesis was first proposed following receptor code analysis for carvone enantiomers [38]. This odor-decoding model considers that the olfactory system can extract sensory information by summating signals from multiple receptors in the third-order neurons of olfactory pathways via input synchronization through feedforward inhibition of the pyramidal cells in the anterior piriform cortex (aPC), the second olfactory center [36,37,39]. This sensory strategy is analogous to that in vision, wherein the four elemental colors (red, green, yellow and blue) are primarily extracted by the third-order neurons (ganglion cells) or the higher visual pathway through summation of synchronized inputs from one or two types of receptors following inhibition by signals from M-cone and S-cone photoreceptors. Elemental colors allow us to perceive all visible hues in different weighted combinations, and similarly, elemental odors likely allow us to discriminate various odors in different weighted combinations.

Olfactory feedforward inhibition is activated in the rostro-ventral portion of the aPC (aPC_vr_) [40]. Notably, in insects, input synchronization via inhibition is also important for discrimination of similar odors [41]. Furthermore, mutual inhibition between different odors was previously examined in a mixture of rose and fox-unique TMT odors in mice [42]. A rose-odor-induced decrease was apparent in cells positive for the TMT odor in the aPC_vr_, and this was accompanied by a subsequent decrease in the TMT-induced stress response. This suggests that signals from ORs activated by the co-applied rose odor weakens the feedforward inhibition from ORs for TMT and thus weakens the subsequent signal integration of cognate TMT ORs. Compared to the sum of the responses to the two individual odors, the total number of cells positive for the mixture of TMT and rose odor in the dorsal part of the anterior piriform cortex also decreased, suggesting a decrease in the perceived intensity of the TMT odor [42]. In contrast to the rose odor, caraway odor did not alleviate the TMT-induced stress response, suggesting a hierarchy of elemental odors in the order rose > TMT > caraway, at least under the experimental conditions employed. Signals from helix-8-second-Glu dorsal ORs are likely associated with the most prominent (upper level) signaling for a given odor (the most prominent elemental odor), whereas other ORs are presumably related to lower levels (auxiliary) of the odor (weaker elemental odors).

In this way, helix-8-second-Glu of ORs appear to govern, at least in part, olfactory information processing of hierarchical elemental odors through earlier and more intense signals than those processed by helix-8-second-Ala or Lys ORs in parallel GPCR signaling pathways. Among 374 (52 class I and 322 class II) human ORs, a total of 45% (23% (12/52) in class I and 48% (155/322) in class II) have a Glu at the second position of helix 8, while the 33% and 16% have an Asp and Gln, respectively (Table 1). Interestingly, Glu and Gln are identical in terms of side-chain size (i.e., they are isosteric). However, although Glu and Asp both have a negative charge, the side chain of Asp is shorter by one carbon atom, and there are no helix-8-second-Asp ORs among human or murine class-I ORs that are expressed in the dorsal zone (dorsal ORs). Moreover, the frequency of helix-8-second-Glu, Asp and Gln ORs are almost identical between human and murine class-I ORs: 23% vs. 24%, 0% vs. 0% and 69% and 67%, respectively (Table 1). Helix-8 second residues were >90% (39/42 and 204/226 in class I and II, respectively) identical between human and murine ORs (Table S1). These results suggest that ORs with different residues at the second position of helix 8 play distinct roles in elemental odor representation. As described above, our results suggest that the most sensitive helix-8-second-Glu dorsal ORs emphasize (*R*)-(−)-carvone-unique elemental odors in the brain by selective summation of cognate OR signals via synchronized inputs to the third-order neurons through feedforward inhibition driven by signals from the most sensitive helix-8-second-Glu dorsal ORs with a shorter onset latency (one of which is enclosed by the red rectangular in Figure 5) [36,37]. If ORs with longer onset latencies determine the most prominent elemental odors, odor perception must change during development as odor representation in the brain adapts over time.

Furthermore, helix-8-second-Glu ORs accounted for 73% (11/15) of the 15 identified carvone ORs in a single-cell RT-PCR study of 2740 randomly sampled murine olfactory sensory neurons, which is 1.6-fold more than the average number of human helix-8-second-Glu ORs. Along with the absence of any helix-8-second-Asp class-I ORs in both human and mouse, this indicates that helix-8-second-Glu ORs operate as determinants of odor representation. Interestingly, helix-8-second-Gln class-I ORs make up the largest group (67%–69%), which is ca. 3-fold and 10-fold larger than helix-8-second-Glu class-I ORs and helix-8-second-Gln class-II ORs, respectively. Future research should focus on the question of which target-prominent or auxiliary elemental odors different types of ORs contribute to identifying, amplifying or classifying.

## 7. Potential Roles of Helix 8 in GPCR Membrane Surface Expression, Internalization, Regulation of Phosphorylation and Dimerization

In parallel GPCR signaling pathways, a proper ratio of signaling proteins is likely required for ensuring adequate sensory information processing or systematic functional regulation. Inhibition of GPCRs by phosphorylation of the C-terminal region may disrupt the proper sequence of multistep interactions between GPCRs and their target G proteins, resulting in their removal from the membrane. Non-interactive GPCR mutants must also be removed because they could reduce the total GPCR sensitivity by capturing target agonists, leading to a decrease in the effective agonist concentration. Arrestin-mediated internalization of phosphorylated GPCRs is likely to be one of the regulatory mechanisms employed to maintain the proper sensitized/desensitized GPCR ratio.

In the thyrotropin-releasing hormone receptor (TRHR, specific to G_q/11_), agonist-dependent phosphorylation by GPCR kinases occurs in both wild type (>35%) and helix-8-second K326R mutant forms (ca. 40%) but not in the K326Q mutant (ca. 5%) [43]. In total, 70% of wild-type TRHR was internalized following complex formation with arrestin, but internalization was only 40% for 6K → 6Q mutant (including helix-8-second-Lys). The high internalization rate suggests that wild-type TRHR may be of less importance than the 6K → 6Q TRHR mutant, or overexpressed to a great extent than could be measured accurately. This also suggests that the G protein coupling specificity-determining second residue of helix 8 is also a determinant of receptor phosphorylation and internalization via formation of arrestin-receptor complexes. When Rhod is phosphorylated in the C-terminal region, the conformational dynamics of helix 8 controls binding to arrestin and subsequent arrestin activation during the desensitization process [44]. Enhancement of agonist-induced receptor internalization by a single mutation in helix 8 has also been reported for the human calcitonin receptor-like receptor [10].

In melanin-concentrating hormone receptor 1, a proximal dibasic pair of residues in the fourth and fifth positions of helix 8 is required for GPCR cell surface expression and signaling [45]. Furthermore, in the type 1 angiotensin receptor, helix 8 has been reported to interact with a myriad of proteins, including caveolin, angiotensin II type 1 receptor-associated protein, and γ-aminobutyric acid receptor-associated protein in membrane expression, G proteins, phospholipase C, Jak2, calmodulin and SHP-2 in signaling, and regulators in lateral receptor migration, receptor internalization and nuclear transcription factors [46]. Helix 8 also plays a key role in protein/lipid interactions [8,9] and dimerization of receptors [11] or heteroreceptors (fibroblast growth factor receptor 1 and 5-hydroxytryptamine 1A receptor that play an enhancing role in hippocampal plasticity) [47], and is, therefore, responsible for multiple functions in parallel GPCR signaling pathways.

## Figures and Tables

**Table 1:** Frequency of helix-8-second residues in human and mouse olfactory receptors (ORs).

Subclass ORs	Helix-8 Second Residue										
	All	Glu	Gln	Asp	Lys	His	Ala	Pro	Tyr	Val	misc
Human class-I ORs	52	12	36	0	1	1	0	1	0	0	1
	100%	23%	69%	0%	2%	2%	0%	2%	0%	0%	2%
Murine class-I ORs	123	29	83	0	5	0	0	3	0	3	0
	100%	24%	67%	0%	4%	0%	0%	2%	0%	2%	0%
Human class-II ORs	322	155	22	128	6	2	3	0	1	1	4
	100%	48%	7%	40%	2%	1%	1%	0%	0%	0%	1%
Total human ORs	374	167	58	128	7	3	3	1	1	1	5
	100%	45%	16%	34%	2%	1%	1%	0%	0%	0%	1%

## Supplementary Materials

Supplementary materials can be found at www.mdpi.com/1422-0067/17/11/1930/s1.
